# Effect of PM_2.5_ pollution on perinatal mortality in China

**DOI:** 10.1038/s41598-021-87218-7

**Published:** 2021-04-07

**Authors:** Guangqin Li, Lingyu Li, Dan Liu, Jiahong Qin, Hongjun Zhu

**Affiliations:** 1 College of International Trade and Economics, Anhui University of Finance and Economics, Bengbu, 233030 Anhui People’s Republic of China; 2 Institute of Finance and Economics Research, Shanghai University of Finance and Economics, Shanghai, 200433 People’s Republic of China; 3 Centre for Health Economics Research and Evaluation, University of Technology Sydney, Sydney, Australia; 4 School of Physical Education and Sports Science, South China Normal University, Guangzhou, 511436 Guangdong People’s Republic of China

**Keywords:** Environmental economics, Socioeconomic scenarios, Environmental social sciences

## Abstract

Using ArcGIS to analyze satellite derived PM_2.5_ estimates, this paper obtains the average concentration and maximum concentration of fine particulate matter (PM_2.5_) in China's 31 provinces from 2002 to 2015. We adopt fixed effects model and spatial Durbin model to investigate the association between PM_2.5_ and perinatal mortality rates. The results indicate that PM_2.5_ has a significantly positive association with perinatal mortality rates. A 1% increase of log-transformed average concentration and maximum concentrations of PM_2.5_ is associated with 1.76‰ and 2.31‰ increase of perinatal mortality rates, respectively. In spatial econometrics analysis, we find PM_2.5_ has significant spatial autocorrelation characteristics. The concentrations of log-transformed average and maximum PM_2.5_ increase 1% is associated with a 2.49% increase in a 2.49‰ and 2.19‰ increase of perinatal mortality rates, respectively. The potential mechanism is that air pollution has an impact on infant weight to impact perinatal mortality rates.

## Introduction

With the rapid growth of the economy, the environment pollution has become a serious issue in China. According to data of the Ministry of Environmental Protection, the average fine particulate matter (PM_2.5_) concentrations reached 72 µg/m^3^ in 2013. Therefore, 99% of the China’s population lived in areas exceeding the World Health Organization (WHO) Air Quality Guideline of 10 µg/m^3^ PM_2.5_^1,2^. According to the Asian Development Bank (ADB) Annual Report 2012, less than 1% of China’s 500 biggest cities is up to the WHO standards, and seven cities in China list among the ten most polluted cities in the world^3^. China has become one of countries with the highest environmental burden of disease in the world^4^. The Institute for Health Metrics and Evaluation Global of Disease in 2012 estimates that outdoor air pollution contributes to 1.20 million premature deaths in China, and air pollution is the fourth leading cause of premature deaths in 67 risk factors^5^. In addition, outdoor air pollution in China is responsible for 12.34 million deaths and 25 million healthy life-years lost per year. The frequency of severe air pollution events has spurred widespread concern about the environment among citizens and scholars.

A great number of studies find a significant and negative relationship between air pollution and health. The levels of PM_2.5_ in the air are associated with the risk of deaths from all causes^6^. Based on the national database of air pollution and mortality for the 88 largest U.S. cities for the period of 1987–1994, which concluded that previous-day PM_10_ concentrations are positively associated with total mortality in most locations, Dominici et al.^7^ find that the mortality rate increased by 0.5% for every µg/m^3^ increase of PM_10_. Wong et al.^8^ and Fang et al.^9^ come to a similar conclusion in studying the effects of air pollution on mortality in Asia and China. Using prefectural panel data from China, Chen and Chen^10^ find that a 1% increase in gas emission leads to an increase in the number of deaths from respiratory diseases and lung cancer by 0.05‰ and 0.03‰, respectively. Pope et al.^11^ find that each 10 µg/m^3^ elevation in fine particulate air pollution is associated with approximately a 4%, 6%, and 8% increased risk of all-cause, cardiopulmonary and lung cancer mortality, respectively.

The carrier of air pollutants such as PM_2.5_ has been linked to lung and cardiovascular diseases, which increase mortality rates^12–16^. A number of studies investigate mechanisms through which air pollution causes diseases. Kampa and Castanas^17^ find that air pollution has both acute and chronic effects on human health by affecting a number of different systems and organs. Tallon et al.^18^ find that exposures to long-term PM_2.5_ and NO_2_ are associated with decreased cognitive function in a cohort of older Americans. Individuals who experience a stroke or elevated anxiety are more susceptible to the effects of PM_2.5_ on cognition.

There is also an important literature which mainly studies the concentration response curve between air pollution and mortality. Its main conclusion is that the concentration response curve between air pollution and mortality is not linear, but nonlinear. In view of this nonlinear relationship, some scholars estimate the logarithm of air pollution and get the relationship between the growth rate of pollution and mortality^19–21^. Liu et al*.*^22^ estimated the shape of concentration–response curves of ballistic "S" curve between PM_2.5_ and cardiopulmonary disease mortality, which was linear in the range of low to medium PM_2.5_ and flattened in the range of high PM_2.5_. The current literature mainly studies the concentration response curve between adult or child mortality and air pollution, but there is little research on the response curve between perinatal mortality and air pollution. Therefore, based on the existing research gaps, this paper focuses on the concentration response curve between perinatal mortality and air pollution.

There is also a literature to study that the different groups of individuals are affected by air pollution in different ways. Vulnerable population, such as children, are more susceptible to the adverse effects of exposure to air pollution than others are. A number of studies examine the adverse health effects of ambient air pollution on kids. Chay & Greenstone^23^ take the significant drop in the level of air pollution caused by the U.S. economic recession of 1981–1982 as an external shock. They conclude that a 1% reduction in total suspended particulates (TSPs) leads to a 0.35% decline in the infant mortality rate at the county level, implying that 2,500 fewer infants died during 1980–1982 than would have in the absence of the TSPs reductions. Chay and Greenstone^24^ find that after the implementation of the 1970 Clean Air Act in the U.S, the air quality has been improved and infant mortality rate has also decreased to a certain extent. Currie and Neidell^25^ examine the impact of three criteria pollutants on infant death in California over the 1990s. Reductions in carbon monoxide over the 1990s saved approximately 1000 infant lives in California. Currie et al.^26^ find negative association with exposure to CO on infant health.

If a mother during pregnancy is exposed to increased environmental stressors, it could result in an increased risk of fetal growth restriction or a preterm birth, which are strong predictors for infant mortality and morbidity^27,28^. DeFranco et al.^29^ find that exposure to high levels of PM_2.5_ in the third trimester of pregnancy is associated with a 42% increase in stillbirth risk. Faiz et al.^30^ find that the relative odds of stillbirth are associated with interquartile range increases in the mean pollutant concentrations on lag day 2 and lag days 2–6 before delivery.

The above studies demonstrate the relationship between air pollution and infant mortality. There are also some literatures on the relationship between air pollution and perinatal mortality. For example, Woodruff et al.^31^ use 4 million infants born between 1989 and 1991 in the 86 metropolitan statistical areas (MSAs) in the United States to find that the particulate matter is associated with risk of post neonatal mortality; De Medeiros et al.^32^ studied the relationship between traffic-induced air pollution and perinatal mortality rates through case studies; Hackley et al.^33^ study the impact that exposure to air pollution has on the health of a pregnancy and offer suggestions on how to minimize exposures.

In this study, we aim to test the hypothesis that exposure to PM_2.5_ in the air during pregnancy is associated with the perinatal mortality rate. Perinatal mortality is an indicator of mother and child health and may reflect the conditions of reproductive health^34,35^. Using data of China’s provincial level PM_2.5_ concentrations from 2002 to 2015, we adopt both fixed effects model and spatial Durbin model (SDM) to investigate the relationship between PM_2.5_ and the perinatal mortality rates. This paper contributes to the literature in several respects. First, many studies on China examine the association between some pollutants, such as CO, PM_10_, SO_2_ and health; however, few studies investigate the effect of PM_2.5_ on mortality. We enrich existing literature by examining the effect of pollutants on mortality rates. Second, we contribute to literature that examines the association between air pollution and perinatal death rates. The prenatal stage of life is a very sensitive period such that exposure to PM_2.5_ pollutions might have an adverse effect on the development of fetuses. Third, we adopt spatial panel model to analyze the spatial autocorrelation of PM_2.5_ pollutions among Chinese provinces and demonstrate time and space lag association between PM_2.5_ pollutions and health.

The paper is organized as follows. “Methodology” section explains our methodology. “Data” section describes the data. “Results” section presents regression results. “Conclusions and discussion” section is the discussion.

## Methodology

We use fixed effects model and spatial econometrics model to estimate the relationship between PM2.5 and infant mortality rates.

### Fixed effects model

We use the following baseline econometric model:

1 $$mortality_{i,t} = \alpha + \beta \left( {\ln PM_{2.5} } \right)_{i,t - 1} + X^{\prime}_{i,t} \gamma + province_{i} + year_{t} + \varepsilon_{i,t}$$

In the above, *i* and *t* indicate the region *i* and year *t*, respectively; $mortality$ is the perinatal mortality rates; province is the province fixed effects; year represents year fixed effects; and $\varepsilon$ is the random disturbance term (In empirical research, we use clustered standard errors at the province level). $\left( {{\text{ln}}PM_{2.5} } \right)_{i,t - 1}$ is the natural logarithms of one-year-lagged PM_2.5_, β is the estimated coefficient of interest. $X^{\prime}$ represents a vector of control variables, including the total number of health agencies per 10,000 people, the total number of health beds per 10,000 population and gross domestic product (GDP) per capita.

### Spatial econometrics model

Shao et al.^36^ show that PM_2.5_ pollution has significant spatial autocorrelation characteristics, which indicates that the perinatal mortality rates are affected not only by the local PM_2.5_ pollution, but also the neighborhood PM_2.5_ pollution. Therefore, we use a spatial econometric approach to investigate the relationship between PM_2.5_ and the perinatal mortality rates.

The most commonly used spatial econometric models in applied research are the spatial lag model (SLM), the spatial error model (SEM) and the SDM model^37^. The SDM model includes spatial lag terms from dependent variables and independent variables to capture the spillover effects deriving from different variables, which is used widely in environment research^38,39^.

We estimate the following SDM specification:

2 $$\begin{aligned} mortality_{i,t} & = \alpha + \rho Wmortality_{i,t} + \beta \left( {{\text{ln}}PM_{2.5} } \right)_{i,t - 1} + \varphi W\left( {{\text{ln}}PM_{2.5} } \right)_{i,t - 1} \\ & \quad + X^{\prime}_{i,t} \gamma + WX^{\prime}_{i,t} \phi + province_{i} + year_{t} + \varepsilon_{i,t} \, \varepsilon_{i,t} \sim (0,\theta^{2} ) \\ \end{aligned}$$

where *W* is the spatial weighting matrix; $Wmortality$ represents perinatal mortality rates in neighboring areas; $W\left( {\ln PM_{2.5} } \right)_{i,t - 1}$ represents the natural logarithms of one-year-lagged PM_2.5_ in neighboring areas; $WX^{\prime}$ is a vector of control variables in neighboring areas; $\rho$ is the spatial autoregressive parameter; $\varphi$ is the coefficient of neighboring PM_2.5_ effecting on local influence; $\gamma$ and $\phi$ are the parameters of the two matrices, respectively; and $\varepsilon$ obeys normal distribution with standard deviation of $\theta \,$.

To study the spatial distribution of perinatal mortality rates in 31 province-level regions, the spatial weight matrix *W* needs to be defined first. There are many specifications for spatial weighting matrix, such as spatial contiguity weights, inverse distance matrix and socio-economic distance matrix, but the most commonly used one is the binary contiguity matrix. In this study, we choose the specification of binary contiguity to create the spatial weight matrix *W.* The elements of spatial weight matrix *W* are defined as *W*_*ij*_ = 1 if location* i* is adjacent to location *j*. It is convenient to normalize spatial weights to remove dependence on extraneous scale factors. Therefore, row-normalized weight matrices are used in the study.

To investigate the spatial clustering pattern of PM_2.5_ and perinatal mortality rates, we calculate Moran’s I index, which is the correlation coefficient of observed values and spatial lagged variables. The value of Moran’s I index is between -1 and 1, with positive values implying positive spatial autocorrelation, negative values implying negative spatial autocorrelation and a zero-value indicating a random spatial pattern. The formula for calculating Moran’s I index is as follows:

3 $$Moran^{\prime}s{\text{ I = }}\frac{{\sum\nolimits_{i = 1}^{n} {\sum\nolimits_{j = 1}^{n} {W_{ij} (x_{i} - \overline{x})(x_{j} - \overline{x})} } }}{{S^{2} \sum\nolimits_{i = 1}^{n} {\sum\nolimits_{j = 1}^{n} {W_{ij} } } }}$$

where $S^{2} = \sum {_{i = 1}^{n} (x_{i} - \overline{x})^{2} /N} , \, \overline{x}{ = }\sum {_{i = 1}^{n} x_{i} /N}$; $x_{i}$ represents mortality rates of region $i$; $N$ is the number of samples; and *W*_*ij*_ is the spatial weighting matrix.

## Data

### Perinatal mortality rates

World Health Organization (WHO) defines a perinatal death as ‘a death occurring at 22 weeks completed weeks of gestation and over, during childbirth and up to seven completed days of life’.

In this paper, the definition of perinatal mortality rate is the ratio of neonatal mortality (including stillbirths) from 28 weeks of gestation to 7 days after delivery to live births (Unit is ‰). Data on perinatal mortality rates is from *China Health and Family Planning Statistical Yearbook*.

### Main explanatory variable (PM_2.5_)

In China, the main sources of air pollution data are data on (TSPs (before 2013 and Air Quality Index (AQI, after 2013). The TSP is a comprehensive index, with only a few cities as monitoring cities. The AQI level is based on the level of six atmospheric pollutants, which covers most major cities in China, but no data is available before 2013. We downloaded the data from the Socioeconomic Data and Applications Center, hosted by the Center for International Earth Science Information Network (CIESIN) at Columbia University. Accordance to research of Van Donkelaar et al.^40,41^, the data is a hybrid product with inputs including Aerosol Optical Depth measured by satellites and a chemical transport modeling component that uses baseline emissions data to model the movement of pollution. The dataset contains information on three-year running mean of PM_2.5_ concentrations for 0.01° × 0.01° grids from 1998 to 2016. Adjacent grid points are approximately 10 km apart. We use ArcGIS software to extract PM_2.5_ estimates data of years from 2002 to 2015. For each province-year observation, we calculate the average and maximum PM_2.5_ concentration using the data of the grid points that fall within the province^42^. We take the average value of PM_2.5_ concentrations as province's annual air pollution level. It should be pointed out that the satellite data in the monitoring process will be affected by meteorological factors, which is slightly lower than the actual ground monitoring data. However, compared to the ground monitoring using "point to surface" measure, the satellite data is relatively reasonable. Therefore, we perform a robustness test using maximum PM_2.5_ concentrations as the air pollution measure. Referring to method of Van Donkelaar et al.^41,42^, Hammer et al.^43^ re-estimate the PM_2.5_ data concluding the provinces, cities and counties data in China from 2000 to 2018, which can be obtained from Atmospheric Composition Analysis Group in Dalhous University. This data is used to test the robustness.

### Other explanatory variables

According to the literature^44^, we control the following variables.

Regional medical conditions. We control for the number of hospital beds per ten thousand persons (*bed_pop*) and the number of hospital agencies per ten thousand persons (*ha_pop*) at the province level, which represent the availability of health care.

Regional economic development level. Regional economy provides the necessary material and nonmaterial support for decreasing perinatal mortality. We assume the higher the level of regional economic development is, the larger the health care spend is. So, a negative relationship between regional economic development and perinatal mortality is expected. In this paper, per capita actual gross domestic product (GDP) is used as a proxy for regional economic development, which is inflation-adjusted by constant 2002 prices.

Urbanization rate. It is the proportion of population in urban areas in a province. It is related to the social economic status (SES) of citizens, such as economic status and education. The higher the urban rate is, the higher the SES of citizens is, and the more attention will be paid to health, resulting in lower perinatal mortality rates. However, higher urban rate will also lead to serious air pollution, which might have a negative effect on perinatal mortality rates.

The above data are available from *China Statistical Yearbook*, *China Health and Family Planning Statistical Yearbook, China Urban Statistical Yearbook* and *China Regional Economic Statistical Yearbook*.

### Descriptive statistics

**Figure 1:**
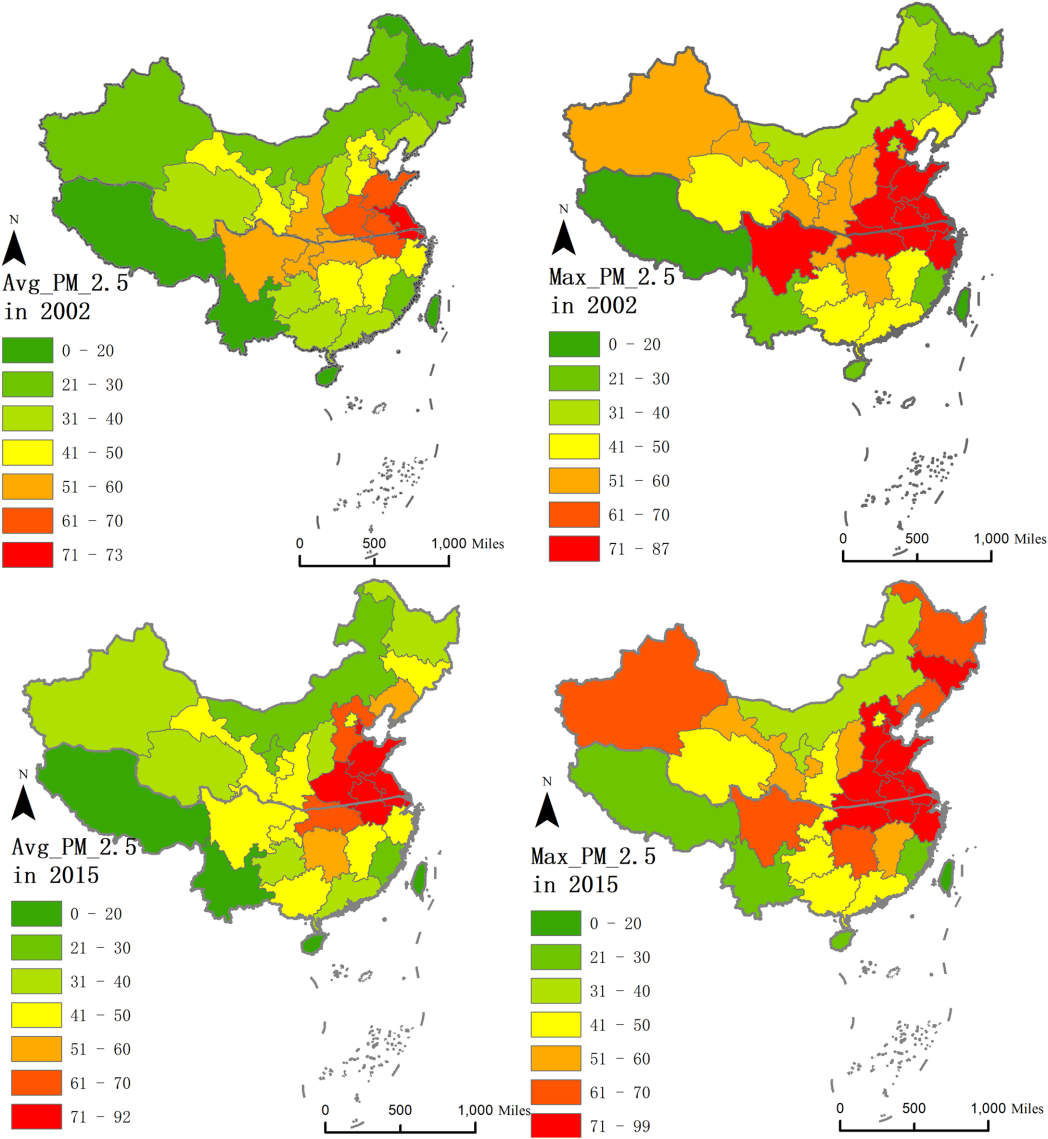
Spatial distribution of PM_2.5_ in 2002 and 2015 (µg/m^3^), respectively. The left two maps are the spatial distribution maps of average PM_2.5_ pollution concentration in 31 provinces in 2002 and 2015, respectively, and the right two maps are the spatial distribution maps of maximum PM_2.5_ pollution depth in 31 provinces in 2002 and 2015, respectively. Maps generated in ArcGIS 10.3.

We construct data of China’s 31 provinces from 2002 to 2015. The descriptive statistics of data is provided in Table 1. Figure 1 shows the spatial distribution of PM_2.5_ in 2002 and 2015 (µg/m^3^). The maximum and average value of PM_2.5_ are higher than Air Quality Guideline (10 µg/m^3^) of the WHO.

**Table 1:** Descriptive statistics for the variables.

Variable	Grouping	Mean	SD	Min	Max	Observations
Perinatal mortality rates (‰)	Overall	9.129	4.547	2.150	25.800	N = 434
	Between		3.618	2.876	20.935	n = 31
	Within		2.824	2.215	20.538	T = 14
Average PM_2.5_ (log)	Overall	3.745	0.484	2.183	4.519	N = 434
	Between		0.479	2.351	4.391	n = 31
	Within		0.108	3.186	4.208	T = 14
Max PM_2.5_ (log)	Overall	3.987	0.416	2.666	4.718	N = 434
	Between		0.405	2.890	4.544	n = 31
	Within		0.117	3.430	4.578	T = 14
New Average PM_2.5_ (log)	Overall	3.500	0.581	1.462	4.426	N = 434
	Between		0.573	1.650	4.256	n = 31
	Within		0.141	2.946	4.055	T = 14
New Max PM_2.5_ (log)	Overall	4.288	0.339	3.114	5.956	N = 434
	Between		0.298	3.255	4.775	n = 31
	Within		0.170	3.863	6.038	T = 14
GDP per capita(log)	Overall	4.701	0.672	2.790	6.107	N = 434
	Between		0.478	3.813	5.671	n = 31
	Within		0.480	3.492	5.618	T = 14
bed_pop	Overall	35.675	10.809	15.318	63.671	N = 434
	Between		6.171	27.839	49.940	n = 31
	Within		8.939	21.434	61.823	T = 14
ha_pop	Overall	5.038	3.446	1.231	21.789	N = 434
	Between		1.957	1.697	12.439	n = 31
	Within		2.856	− 2.860	14.388	T = 14
Urbanization rate (%)	Overall	48.327	15.252	13.890	89.600	N = 434
	Between		13.112	22.351	84.071	n = 31
	Within		8.115	− 11.190	85.220	T = 14

## Results

### Fixed effects model

#### Baseline model

Fixed effects model is used based on the Hausman test. Table 2 displays the results of the baseline model (1). Column (1) and Column (2) reports the results of the impacts of average concentrations of PM_2.5_ on perinatal mortality rates controlling for only province fixed effects or both province and year fixed effects. Column (3) and (4) include all controls on the basis of the first two columns. The estimate coefficients of the log value of *PM*_*2.5*_ are 2.439, 2.759, 1.644 and 1.76, respectively, which are positive and significant. Column (4) is our referred model, which shows that for every 1% increase in (log) pollution, we find an associated 1.76% increase in perinatal mortality rates.

**Table 2:** The impacts of average PM_2.5_ concentrations on perinatal mortality rates.

Explanatory variable	Explained variable: *Perinatal mortality*				
	(1)	(2)	(3)	(4)	(5)
Average PM_2.5_ (log)	2.439***	2.759***	1.644**	1.760**	
	(0.749)	(0.957)	(0.751)	(0.886)	
Max PM_2.5_ (log)					2.312***
					(0.770)
GDP per capita(log)			− 3.644***	− 5.161***	− 5.050***
			(0.389)	(1.100)	(1.094)
bed_pop			− 0.073***	− 0.076***	− 0.073***
			(0.018)	(0.027)	(0.027)
ha_pop			− 0.065	− 0.106*	− 0.110**
			(0.041)	(0.054)	(0.054)
Urbanization			0.006	− 0.001	0.001
			(0.013)	(0.013)	(0.013)
Constant	24.009***	34.874***	22.769***	28.819***	25.696***
	(2.650)	(4.885)	(2.632)	(5.233)	(5.127)
Year fixed effects	N	Y	N	Y	Y
Province fixed effects	Y	Y	Y	Y	Y
Within R-squared	0.685	0.691	0.702	0.700	0.704
Observations	434	434	434	434	434

Standard errors in parentheses. *p < 0.1, **p < 0.05, ***p < 0.01.

The association with the average PM_2.5_ concentrations and the perinatal mortality in the Column (4) might be underestimated because of measurement error. Therefore, we define maximum concentrations of PM_2.5_ as a measure of PM_2.5_ pollutions for re-estimate the association. The results are presented in Table 3. After controlling covariates, the results in column (5) show that maximum concentrations of PM_2.5_ have a significant and positive association with the perinatal mortality rates. A 1% increase of the log value of maximum concentrations of PM_2.5_ is association with 2.312‰ increase of perinatal mortality rates. The association with the maximum PM_2.5_ concentrations and the perinatal mortality is stronger.

**Table 3:** Robustness check.

Explanatory variable	Explained Variable: *Perinatal mortality*					
	(1)	(2)	(3)	(4)	(5)	(6)
New Average PM_2.5_ (log)	1.127*				1.139**	
	(0.621)				(0.451)	
New Max PM_2.5_ (log)		1.222**				1.374***
		(0.487)				(0.342)
Average PM_2.5_ (log)			1.035*			
			(0.621)			
Max PM_2.5_ (log)				1.758***		
				(0.554)		
GDP per capita(log)	− 5.133***	− 4.802***	− 2.847**	− 2.834**	− 2.876**	− 2.449*
	(1.082)	(1.099)	(1.378)	(1.380)	(1.370)	(1.398)
bed_pop	− 0.030	− 0.038	0.149***	0.150***	0.141***	0.149***
	(0.031)	(0.031)	(0.044)	(0.044)	(0.044)	(0.044)
ha_pop	− 0.066***	− 0.070***	− 0.075**	− 0.077**	− 0.073**	− 0.079**
	(0.024)	(0.024)	(0.035)	(0.035)	(0.035)	(0.035)
Urbanization	− 0.017	− 0.009	− 0.141	− 0.134	− 0.150*	− 0.136
	(0.063)	(0.063)	(0.088)	(0.088)	(0.088)	(0.088)
Constant	28.681***	30.520***	27.426***	26.596***	27.877***	20.730***
	(5.056)	(4.735)	(5.703)	(5.691)	(5.396)	(5.535)
Year fixed effects	Y	Y	Y	Y	Y	Y
Province fixed effects	Y	Y	Y	Y	Y	Y
Province*Time trend			Y	Y	Y	Y
Within Rsquared	0.737	0.741	0.897	0.897	0.898	0.897
Observations	434	434	434	434	434	434

Standard in parentheses, *p < 0.1, **p < 0.05, ***p < 0.01.

The coefficient of the GDP (log) per capita is -5.161 in Column (4), which is negative at the 1% significance level, meaning that a 1% increase of the log value of GDP per capita results in 5.16‰ increase of perinatal mortality rates. The coefficient of *bed_* number of hospital beds per ten thousand persons and the number of hospital agencies per ten thousand persons *are* -0.076 and -0.106 respective, which mean they and are negatively related to perinatal mortality rates (shown in Column (4)), which shows that the improvement of access to healthcare is conducive to reducing mortality rates. The coefficients of urbanization rates are insignificant.

#### Robustness check

Considering the PM_2.5_ data updated by hammer et al.^44^, this paper uses the updated PM_2.5_ data for estimation, and the estimation results are presented in column (1) and column (2) of Table 3. The results show that the estimated coefficient of the new PM_2.5_ data has a certain decline, but it is still significantly positive, indicating that the positive association with PM_2.5_ pollution and the perinatal mortality is still valid.

In order to control for time-varying, unobserved characteristics at the province-level to some extent, the columns (3)–(6) of the Table 3 are the results under controlling the interaction between provinces and time trend to test the robustness. It can be seen from the results that whether using the PM_2.5_ data from Columbia University or the new PM_2.5_ data from Dalhous University, PM_2.5_ pollution can bring to a significant increase in perinatal mortality. The difference between the results and those in Table 2 is mainly reflected in the change of coefficient. Once again, this result is robust, that is, the PM_2.5_ pollution has a significant positive association with the perinatal mortality.

### Spatial analysis

#### Moran’s I index

Table 4 lists the results of the *Moran’s I* test for overall spatial correlation of perinatal mortality rates and regional PM_2.5_. The values of *Moran’s I* for mortality rates are more than 0.3 and pass the 1% significance test, which indicates that there exists significant and positive autocorrelation among regional perinatal mortality rates in the geographical space. There also exists significant positive autocorrelation among regional PM_2.5_.

**Table 4:** The *Moran’s I* test for spatial correlation.

Year	Perinatal mortality		Average PM_2.5_ (log)		Max PM_2.5_ (log)	
	*Moran’s I*	*P*-value	*Moran’s I*	*P*-value	*Moran’s I*	*P*-value
2002	0.426	0.000	0.484	0.000	0.337	0.002
2003	0.463	0.000	0.505	0.000	0.384	0.001
2004	0.558	0.000	0.497	0.000	0.327	0.003
2005	0.623	0.000	0.506	0.000	0.352	0.001
2006	0.600	0.000	0.505	0.000	0.371	0.001
2007	0.560	0.000	0.558	0.000	0.458	0.000
2008	0.477	0.000	0.512	0.000	0.330	0.003
2009	0.396	0.000	0.484	0.000	0.393	0.000
2010	0.414	0.000	0.481	0.000	0.339	0.002
2011	0.478	0.000	0.520	0.000	0.389	0.000
2012	0.348	0.000	0.481	0.000	0.351	0.001
2013	0.386	0.000	0.525	0.000	0.425	0.000
2014	0.392	0.000	0.504	0.000	0.360	0.001
2015	0.403	0.000	0.546	0.000	0.496	0.000
Average	0.466	0.000	0.508	0.000	0.379	0.001

#### Results of spatial analysis

Table 5 reports the results of formula (2). The spatial lag coefficients of perinatal mortality rates were significant and negative in four models, indicating that it is competitive in improving health among neighboring provinces; that is, the decrease of mortality rates in the surrounding provinces would promote the decrease of mortality rate in the region. Column (1) and column (2) present the result using the average concentrations of PM_2.5_. Column (3) and column (4) present the corresponding results using maximum concentrations of PM_2.5_. Column (1) and column (3) are the result only controlling for the province fixed effects. Column (2) and column (4) further control the year fixed effects. The difference between the coefficients of PM_2.5_ in four model is very small, which are all significant at the 5% level.

**Table 5:** Results with spatial Durbin Model.

Explanatory variable	Explained Variable: *Perinatal mortality*			
	(1)	(2)	(3)	(4)
W*Mortality	− 0.125*	− 0.248***	− 0.137*	− 0.263***
	(0.072)	(0.074)	(0.072)	(0.074)
Average PM_2.5_ (log)	2.454**	2.491**		
	(1.161)	(1.112)		
Max PM_2.5_ (log)			2.293**	2.186**
			(0.893)	(0.856)
GDP per capita(log)	− 6.270***	− 5.150***	− 6.165***	− 5.003***
	(0.890)	(0.997)	(0.887)	(0.992)
bed_pop	− 0.055**	− 0.055*	− 0.055**	− 0.056**
	(0.028)	(0.029)	(0.028)	(0.028)
ha_pop	− 0.035	− 0.044	− 0.044	− 0.051
	(0.051)	(0.053)	(0.051)	(0.053)
Urbanization	− 0.000	0.006	0.000	0.007
	(0.012)	(0.012)	(0.012)	(0.012)
W*Average PM_2.5_ (log)	− 1.864	− 1.622		
	(1.542)	(1.687)		
W* Max PM_2.5_ (log)			− 0.967	0.014
			(1.247)	(1.379)
W* GDP per capita(log)	2.300**	7.673***	2.014**	7.398***
	(0.993)	(1.829)	(0.990)	(1.821)
W*bed_pop	− 0.038	− 0.207***	− 0.035	− 0.196***
	(0.034)	(0.059)	(0.034)	(0.058)
W*ha_pop	− 0.087	− 0.420***	− 0.077	− 0.409***
	(0.062)	(0.099)	(0.062)	(0.098)
W* Urbanization	0.040	0.024	0.040	0.028
	(0.029)	(0.030)	(0.029)	(0.030)
Year fixed effects	N	Y	N	Y
Province fixed effects	Y	Y	Y	Y
Within R-squared	0.735	0.543	0.736	0.527
Observations	434	434	434	434

Clustered standard in parentheses, *p < 0.1, **p < 0.05, ***p < 0.01.

Spatial effects can be further decomposed into direct effect, indirect effect (spillover effects) and total effect with reference to the research result of LeSage and Pace^45^. Table 6 illustrates the direct effect, indirect effect and total effect of the variables in SDM model. The results show that the estimated coefficient of PM_2.5_′s direct effect has the same direction as the estimated coefficients of SDM model in Table 6. But the estimated coefficient of PM_2.5_′s indirect effect is negative and insignificant. When it comes to the estimated coefficients of controlled variables, we find that not all the spatial spillover effects of variables are significant. Overall, the GDP per capita have direct and indirect influence on perinatal mortality. The number of hospital beds per thousand persons has the significant direct effect, while the indirect effect is insignificant.

**Table 6:** Decomposition of direct effect, indirect effect, and total effect.

Explanatory variable	Explained Variable: *Perinatal mortality*			
	(1)	(2)	(3)	(4)
**Direct effect**				
Average PM_2.5_ (log)	2.483**	2.580**		
	(1.183)	(1.174)		
Max PM_2.5_ (log)			2.308**	2.194**
			(0.912)	(0.903)
GDP per capita(log)	− 6.360***	− 5.643***	− 6.258***	− 5.512***
	(0.915)	(1.022)	(0.912)	(1.019)
bed_pop	− 0.053*	− 0.044	− 0.054**	− 0.045
	(0.028)	(0.029)	(0.027)	(0.029)
ha_pop	− 0.035	− 0.023	− 0.043	− 0.030
	(0.051)	(0.055)	(0.052)	(0.056)
Urbanization	− 0.001	0.005	− 0.001	0.006
	(0.013)	(0.013)	(0.013)	(0.013)
**Indirect effect**				
Average PM_2.5_ (log)	− 1.960	− 1.885		
	(1.509)	(1.601)		
Max PM_2.5_ (log)			− 1.147	− 0.459
			(1.216)	(1.290)
GDP per capita(log)	2.846***	7.716***	2.623***	7.460***
	(0.978)	(1.533)	(0.973)	(1.515)
bed_pop	− 0.031	− 0.169***	− 0.026	− 0.157***
	(0.033)	(0.050)	(0.032)	(0.049)
ha_pop	− 0.075	− 0.349***	− 0.063	− 0.336***
	(0.061)	(0.084)	(0.061)	(0.083)
Urbanization	0.037	0.019	0.036	0.022
	(0.027)	(0.026)	(0.026)	(0.026)
**Total effect**				
ln*PM*_*2.5*_	0.523	0.695		
	(0.854)	(0.969)		
Max PM_2.5_ (log)			1.162	1.735*
			(0.803)	(0.892)
GDP per capita(log)	− 3.514***	2.074	− 3.635***	1.948
	(0.411)	(1.610)	(0.399)	(1.581)
bed_pop	− 0.084***	− 0.212***	− 0.080***	− 0.202***
	(0.019)	(0.043)	(0.018)	(0.042)
hs_pop	− 0.109**	− 0.372***	− 0.106**	− 0.365***
	(0.045)	(0.081)	(0.044)	(0.079)
Urbanization	0.036	0.024	0.036	0.028
	(0.026)	(0.026)	(0.026)	(0.026)

Clustered standard in parentheses, *p < 0.1, **p < 0.05, ***p < 0.01.

### Mechanisms

In this section, we explore how PM_2.5_ pollution affects perinatal mortality rates. The PM_2.5_ pollutions may affect the ratio of infants weighing less than 2.5 kg. Infants weighing less than 2.5 kg are considered as low birth weight, who have a higher risk of early childhood death. The proportion of infants with low birth weight in a province (*w5*) is obtained to investigate whether it is the mechanism through which PM_2.5_ pollution has an impact on perinatal mortality rates. The results are presented in column (1) and (2) of Table 7. An interaction of w5*average PM_2.5_ (log) is included column (1) and w5*Max PM_2.5_ (log) is included in column (2). We find that the coefficients of PM_2.5_ pollutions and *w5* were not significant, only the coefficients of the interactions are positive and statistically significant. There are two reasons for this result: first, due to the serious PM_2.5_ pollutions, the weight of perinatal infants significantly is affected, bring to a larger proportion of perinatal infants less than 2.5 kg, increasing their risk of death. Secondly, because the weight of perinatal infants is lower (less than 2.5 kg), they live in the province with more serious PM_2.5_ pollutions, which will increase their risk of death. But no matter what the possible reason is, PM_2.5_ pollution and weight together affect the perinatal mortality.

**Table 7:** Estimated results of mechanism analysis.

Explanatory variable	Explained variable: *Perinatal mortality*	
	(1)	(2)
Average PM_2.5_ (log)	0.433	
	(1.307)	
w5* Average PM_2.5_ (log)	0.626**	
	(0.265)	
Max PM_2.5_ (log)		0.807
		(0.939)
w5* Max PM_2.5_ (log)		0.652**
		(0.268)
*w5*	− 1.085	− 1.387
	(0.922)	(0.977)
*Urbanization*	− 0.003	− 0.000
	(0.019)	(0.018)
GDP per capita(log)	− 6.034***	− 5.766***
	(1.912)	(1.934)
*bed_pop*	0.021	0.012
	(0.055)	(0.054)
*ha_pop*	− 0.040	− 0.039
	(0.042)	(0.039)
*Constant*	33.247***	30.869***
	(6.468)	(6.664)
Year fixed effects	Y	Y
Province fixed effects	Y	Y
Within Rsquared	0.773	0.774
Observation	434	434

Standard errors in parentheses. *p < 0.1, **p < 0.05, ***p < 0.01.

## Conclusions and discussion

The air quality in China, particularly the PM_2.5_ level, has become an increasing public concern because of its relation to health risks. Using ArcGIS to analyze satellite raster estimates data, this paper explores the relationship between PM_2.5_ pollution and perinatal mortality rates in China for the years of 2002–2015. The main results are as follows: (1) The PM_2.5_ pollution has a significant and positive association with the perinatal mortality rates. A 1% increase of average or maximum concentrations of PM_2.5_(log) bring to 1.76‰ increase of perinatal mortality rates. These conclusions are similar to those of the study on air pollution and infant mortality rate and child mortality rate^31–33^. (2) The PM_2.5_ pollution has strong spatial dependence after analyzing Moran’s I index of the PM_2.5_ pollutions. Therefore, we apply SDM method and find local and neighborhood PM_2.5_ pollution has a significant and positive impact on local perinatal mortality rates. A possible explanation is that pollutants move between areas due to natural conditions such as rainfalls, and wind. (3) The mechanisms analysis showed that PM_2.5_ pollutions would affect perinatal mortality rates through the weight of newborn infants.

This paper contributes to the literature linking PM_2.5_ pollutions to perinatal mortality rate as there has been very little empirical evidence. It also provides policy-making basis for government to put more efforts to prevent and control PM_2.5_ pollutions. The policy recommendations of this paper are as follows: Firstly, the state should increase investment to control the PM_2.5_ pollutions, and improve the efficiency of primary energy utilization for reducing the generation of PM_2.5_ pollutant emissions; Secondly, the state should promulgate relevant laws and regulations to strengthen joint prevention and control of air pollution among regions; Thirdly, pregnant women should try to be exposed to as little pollution as possible. For example, they could install air purifier indoors or wear a mask when going out.

There are still some deficiencies in this study. Firstly, China is a typical country with urban–rural dual structure. Because of the limitations of data, it is impossible to conduct analysis for urban and rural areas separately in the paper. Secondly, this paper uses on macro-data, but micro-data may better identify the relationship between PM_2.5_ pollutant emissions and perinatal mortality rates. Thirdly, this paper only studies PM_2.5_ pollutions, which can be expanded about the impact of other pollutants on perinatal mortality rates. Fourthly, due to the missing variables, this paper can’t identify the causality between PM_2.5_ pollutant emissions and perinatal mortality rates, only get the association between them.
